# Advances in Wood Composites

**DOI:** 10.3390/polym12010048

**Published:** 2019-12-30

**Authors:** Antonios N. Papadopoulos

**Affiliations:** Laboratory of Wood Chemistry and Technology, Department of Forestry and Natural Environment, International Hellenic University, GR-661 00 Drama, Greece; antpap@for.ihu.gr

Wood composites are manufactured from a variety of materials. They usually contain the same woods that are used in lumber, but they are combined to make them stronger and more durable. Wood has long been used as a construction material and revered for its strength and natural aesthetics. However, with forests being chopped down across the globe to meet our insatiable demands, it’s time to look towards an alternative solution and wood composite may be the answer. Wood composites include a range of different derivative wood products, all of which are created by binding the strands, fibers, or boards of wood together. It’s also known as manmade wood, manufactured board or engineered wood, as well as wood-plastic composite (WPC) when using wood fibers and thermoplastics. Similar composite products can also be made from vegetable fibers using lignin-containing materials such as hemp stalks, sugar cane residue, and rye and wheat straw, with chemical additives enabling the integration of polymer and wood flour while helping facilitate optimal processing conditions. They are fixed using adhesives and are engineered to certain specifications, resulting in a material that can have diverse applications. The best part about wood composites is that they can be created using wood waste materials and smaller trees, reducing the need to fell old-growth forests. Composite wood products can be used in a variety of different ways, including both home and industrial construction, and are often used to replace steel for joists and beams in building projects. Their most widespread use, however, is in outdoor deck flooring, but they are also popular for railings, fencing, benches, window and door frames, cladding, and landscaping work. While composite wood can be used in most applications traditionally using solid wood, it is also a popular material for making flat-pack furniture due to its low manufacturing costs and light weight properties.

One of the main advantages of wood composite is that because it is man-made, it can be designed for specific qualities or performance requirements. It can be made into different thicknesses, grades, sizes and exposure durabilities, as well as manufactured to take advantage of the natural strength characteristics of wood (and sometimes results in a greater structural strength and stability than regular wood). As a result, it can be used in a diverse array of applications, from industrial scale to small home projects, and enable more design options without sacrificing structural requirements. Wood composites have also some disadvantages. Some of these, does require more primary energy for its manufacture when compared to solid lumber. Some particle and fiber-based composite woods are also not suitable for outdoor use as they can absorb water and be more prone to humidity-induced warping than solid woods. Another concern regarding wood composites is the adhesives used in their design with some resins releasing toxic formaldehyde in the finished product (particularly those made with urea-formaldehyde bonded products which is one of the cheapest and most common adhesives). The plastic materials often used in the creation of wood composites also have a higher fire hazard when compared to solid wood products, due to their higher chemical heat content and melting properties. With the widespread use of wood composites in the modern world—from panel products to engineered lumber—there is a need to understand their strengths and weaknesses with respect to weathering and decay.

Wood composites have shown very good performance, and substantial service lives when correctly specified for the exposure risks present. Selection of an appropriate product for the job should be accompanied by decisions about the appropriate protection, whether this is by design, by preservative treatment or by wood modification techniques. In this Special Issue, Advances in Wood Composites presents recent progress in enhancing and refining the performance and properties of wood composites by chemical and thermal modification and the application of smart nanomaterials, which have made them a particular area of interest for researchers. In addition, it reviews some important aspects in the field of wood composites, with particular focus on their materials, applications, and engineering and scientific advances, including solutions inspired biomimetrically by the structure of wood and wood composites. This Special Issue, with a collection of 13 original contributions, provides selected examples of recent Advances in Wood Composites.

The main drawbacks of wood, namely susceptibility to biodegradability by microorganisms and a dimensional instability when subjected to a varied moisture content, are mainly due to the cell wall main polymers and, in particular, due to their high abundance of hydroxyl groups (OH) [1,2,3]. This is solved, so far, either by using imported tropical woods or by using conventional biocides which are usually based in the use of toxic chemicals. In addition, the disposal of the treated wood has caused many restrictions which have to be taken into consideration upon the utilization of conventional chemical treatments. However, both these options are nowadays under political and consumer pressure and alternatives have become an imperative need [2]. This need led to attention to non-biocide treatments, such as the chemical and thermal modification of wood, and inorganic modification by sol-gel technologies.

Acetylation and in situ polymerization are two typical chemical modifications that are used to improve the dimensional stability of lignocellulosic materials, like bamboo. The combination of chemical modification of vinyl acetate (VA) acetylation and methyl methacrylate (MMA) in situ polymerization of bamboo was investigated [4]. Performances of the treated bamboo were evaluated in terms of dimensional stability, wettability, thermal stability, chemical structure, and dynamic mechanical properties. The results revealed that the dimensional stability of bamboo after the combined treatment of VA and MMA was remarkably improved because of the decrease in hydrophilic hydroxyl groups. VA and MMA were mainly grafted onto the surface of the cell walls or in the bamboo cell lumen. From TG analysis, an increase in the extent of pretreatment via chemical modifications resulted in the peak temperatures of the maximum weight loss gradually moving toward the side of higher pyrolysis temperature.

Thermal modification is an ecological and low-cost pretreatment method and it is normally performed at between 160 to 260 °C in a vacuum, nitrogen, air, or oil environments [5,6]. A recent study investigated the relevance between the evolution of chemical structure and the physical and mechanical properties during wood thermal modification processes [7]. Moreover, the volatility of compounds (VOCs) was analyzed using a thermogravimetric analyzer coupled with Fourier transform infrared spectrometry (TGA-FTIR) and a pyrolizer coupled with gas chromatography/mass spectrometer (Py–GC/MS). The designed temperatures for heat treatment were 160, 180, and 200 °C and the durations were 3, 6, and 9 h. The results indicated that the dimensional stability improved markedly due to the reduction of hydrophilic hydroxyl groups. Based on a TGA-FTIR analysis, the small molecular gaseous components were composed of H_2_O, CH_4_, CO_2_, and CO, where H_2_O was the dominant component with the highest absorbance intensity. Based on the Py–GC/MS analysis, the VOCs were shown to be mainly composed of acids, aldehydes, ketones, phenols, furans, alcohols, sugars, and esters, where acids were the dominant compounds.

Among the sol-gel technologies, the sol-gel derived wood-inorganic composite (WIC) approaches have received a lot of attention over the last few years [8,9,10]. These WICs have been proven to be effective in improving the flame retardancy, thermal stability, UV stability, and fungal resistance compared to wood. In an interesting study, methyltrimethoxysilane (MTMOS), methyltriethoxysilane (MTEOS), tetraethoxysilane (TEOS), and titanium (IV) isopropoxide (TTIP) were used as precursor sols to prepare wood-inorganic composites [11]. (WICs) by a sol-gel process, and subsequently, the long-term creep behavior of these composites was estimated by application of the stepped isostress method (SSM). The results revealed that the flexural modulus of wood and WICs were in the range of 9.8–10.5 GPa, and there were no significant differences among them. However, the flexural strength of the WICs was stronger than that of wood. Additionally, based on the SSM processes, smooth master curves were obtained from different SSM testing parameters, and they fit well with the experimental data. These results demonstrated that the SSM was a useful approach to evaluate the long-term creep behavior of wood and WICs. Furthermore, the WICs exhibited better performance on the creep resistance than that of wood, except for the WICMTEOS. The reduction of time-dependent modulus for the WIC prepared from MTMOS was 26% at 50 years, which is the least among all WICs tested. These findings clearly indicate that treatment with suitable metal alkoxides could improve the creep resistance of wood.

Another option to overcome the main drawbacks of wood, namely susceptibility to biodegradability by microorganisms and a dimensional instability when subjected to a varied moisture content, perhaps a more attractive one, is to investigate the potentials that nanotechnology may offer. It is well documented in literature, that the cell wall of wood presents a significant porosity; this porosity is of molecular scale [12]. The main reason of employing nanotechnology in the so called wood science and technology, is the unique characteristic of nano-based materials to penetrate deeply on the wood substrates with an effective way, which in turns results in the alteration altering of its surface chemistry. This subsequently causes an improvement in wood properties [13,14]. Any potential change in the wood properties due to treatment with nanomaterials, is based in the higher interfacial area which is developed due to treatment. This is happened because the amount of particles is significantly reduced to nanoscale. The nanomaterials improve the properties of wood as raw material and alter its original feature to a limited extent. Currently, nano-based materials may be effectively applied to wood through the following distinctive ways: (a) through impregnation of nano-based materials, (b) as a polymeric nanocarriers through impregnation of nano-based materials, and (c) as coatings [14,15,16,17].

Bayani et al. [18] investigated the physical and mechanical properties of thermally modified beech wood impregnated with silver nano-suspension and examined their relationship with the crystallinity of cellulose. Specimens were impregnated with a 400 ppm nanosilver suspension. Heat treatment took place in a laboratory oven at three temperatures, namely 145, 165, and 185 °C. Physical properties and mechanical properties of treated wood demonstrated statistically insignificant fluctuations at low temperatures compared to control specimens. On the other hand, an increase of temperature to 185 °C had a significant effect on all properties. As a consequence of the thermal modification, part of amorphous cellulose was changed to crystalline cellulose. At low temperatures an increased crystallinity caused some of the properties to be improved. Change of amorphous cellulose to crystalline cellulose, as well as cross-linking in lignin, partially ameliorated the negative effects of thermal degradation at higher temperatures and therefore, compression parallel to grain and modulus of elasticity did not decrease significantly. Previous studies reported increased thermal conductivity in solid wood and wood-based composites with the addition of nano-metals and nano-minerals [19,20]. The increased thermal conductivity ultimately caused higher degradation in the main wood components and therefore, mechanical properties and pull-off strength decreased in specimens thermally modified at temperature higher than 165 °C. It was concluded that impregnating specimens with silver nano-suspension prior to thermal modification enhanced the effects of thermal modification as a result of improved thermal conductivity [18].

Wollastonite (a silicate mineral, CaSiO_3_) makes bonds with cell-wall polymers of wood. Formation of these bonds results in significant improvement in physical and mechanical properties in composite panels treated with nanowollastonite suspension [21]. Moreover, high thermal conductivity coefficient of wollastonite, along with its non-combustible mineral nature, was reported to ultimately cause improvement in fire properties [22]. Hassani et al. [23] examined the effect of fortification level of nanowollastonite on UF resin and its effect on the mechanical and physical properties of oriented strand lumber (OSL). Two resin contents were applied, namely 8% and 10%. Nanowollastonite was mixed with the resin at two levels of 10% and 20%. It was found that that the fortification of UF resin with 10% nanowollastonite can be considered as an optimum level. When nanowollastonite content was higher (that is, 20%), higher volume of UF resin was absorbed by wollastonite nanofibers, being left from the process of sticking the strips together. The mechanism involved in the fortification of UF resin with nanowollastonite, which resulted in of thickness swelling values can be attributed to the following two factors: (i) nanowollastonite compounds made active bonds with the cellulose hydroxyl groups, putting them out of reach for the water molecules to make bonds with these, and (ii) high thermal conductivity coefficient of wollastonite improved the transfer of heat to different layers of OSL mat, facilitating better and more complete resin curing. Since nanowollastonite contributes in making bonds between the wood strips and consequently improves physical and mechanical properties, its use can be safely recommended in the OSL production process to improve physical and mechanical properties of the panel.

The adhesives used in the manufacture of wood composites have arguably the most influence on the composites properties. The adhesives influence all aspects of the composites, from their mechanical properties and their ability to perform in wet conditions to their effects on the environment (both localized and the wider environment). Composites industry uses almost solely petrol-based adhesives, such as urea-formaldehyde and melamine-urea-formaldehyde. There are two main drivers to replace these synthetic systems with formaldehyde-free bio-based alternatives; lowered formaldehyde limits and the need for sustainability. Bio-based adhesives can be fully formaldehyde-free and have the additional benefit of increased sustainability. However, to reach 100% bio-based formulations is challenging, and the focus has been on increasing the content of bio-based material with a stepwise approach [24].

The most researched biomaterial for wood panel adhesives is lignin. One of the main uses of lignin residues is lignin-phenol-formaldehyde resins, where lignin is used to partially replace phenol. The phenol replacement amounts are typically below 50%, as the addition of lignin lowers the reactivity of the resin, leading to increased reaction times [25]. The utilization of proper crosslinker is an important factor for developing a lignin-based adhesive that can meet the reaction speed and adhesion strength requirements of the wood panel industry. In a recent study [26], two crosslinkers were tested for ammonium lignosulfonate (ALS)—bio-based furfuryl alcohol (FOH) and synthetic polymeric 4,4′-diphenylmethane diisocyanate (pMDI). The derived ALS adhesives were used for gluing 2-layered veneer samples and particleboards. Differential scanning calorimetry showed a reduction of curing temperature and heat for the samples with crosslinkers. Light microscopy showed that the FOH crosslinked samples had thicker bondlines and higher penetration, which occurred mainly through vessels. Tensile shear strength values of two-layered veneer samples glued with crosslinked ALS adhesives were at the same level as the melamine reinforced urea-formaldehyde (UmF) reference. For particleboards, the FOH crosslinked samples showed a significant decrease in these mechanical properties: internal bond (IB), modulus of elasticity (MOE), modulus of rupture (MOR), and thickness swelling. For pMDI crosslinked samples, these properties increased compared to the UmF. Although the FOH crosslinked ALS samples can be classified as non-added-formaldehyde adhesives, their emissions were higher than what can be expected to be sourced from the particles.

In order to reduce the cost of plywood and save edible resources (wheat flour), a cheap and resourceful clay, sepiolite, was used to modify urea formaldehyde (UF) resin [27]. The performances of filler-filled UF resins were characterized by measuring the thermal behavior, cross section, and functional groups. Results showed that cured UF resin with SEP (sepiolite) formed a toughened fracture surface, and the wet shear strength of the resultant plywood was maximum improved by 31.4%. The tunnel structure of SEP was beneficial to the releasing of formaldehyde, as a result, the formaldehyde emission of the plywood bonded by UF resin with SEP declined by 43.7% compared to that without SEP. This study provided a new idea to reduce the formaldehyde emission, i.e., accelerating formaldehyde release before the product is put into use.

Bekhta and Sedliacik [28], presented the first effort to develop and evaluate composites based on alder veneers and high-density polyethylene (HDPE) film, since it was known that thermoplastic films exhibit good potential to be used as adhesives for the production of veneer-based composites. Three types of adhesives were used: urea-formaldehyde (UF), phenol-formaldehyde (PF), and HDPE film. UF and PF adhesives were used for the comparison. The findings of this work indicate that formaldehyde-free HDPE film adhesive gave values of mechanical properties of alder plywood panels that are comparable to those obtained with traditional UF and PF adhesives, even though the adhesive dosage and pressing pressure were lower than when UF and PF adhesives were used. It was concluded that environmentally-friendly high-density polyethylene-bonded formaldehyde-free alder plywood panels have been successfully produced using thermoplastic polymers as an adhesive.

The majority of wood composites in their final use, contain additives. These may include protective coatings, coatings to improve its aesthetic appearance, preservatives for protection against fire or biological factors like fungi and insects, and even plastics to successfully create new types of products [29]. When wood is used in outdoor applications, its surface is very much exposed to many agents and its protection is an imperative need. Its protection therefore can be achieved with an efficient manner, which includes coating, this can be done with surface or bulk treatment. Coatings, which are currently applied to wood surface can be categorized as air-drying coatings, reaction-curing coatings, water-soluble coatings, stains, and oils and waxes [16,29]. A fast water-based ultraviolet light (UV) curing polyurethane-acrylate (PUA) wood coating was prepared and applied on oak (*Quercus alba* L.) at different coating amounts [30]. The coating amounts affected the coating properties after curing on oak. With the increase of coating amount, the adhesion, hardness, and gloss value of the surface increased to different extents. Meanwhile, the surface of sample became smooth gradually because the voids of the oak were filled. Thus, higher coating amount resulted in better coating properties. However, no significant increase of penetration depth was found. During curing, the hydroxyl groups of the wood reacted with the coating. The optimal parameter in this study was the coating amount of 120 g/m^2^, where the adhesion reached 1 (with 0%–5% cross-cut area of flaking along the edges), with the hardness of 2H and the gloss of 92.56°.

Another interesting topic is the preparation of biomorphic porous SiC ceramics from bamboo by combining sol-gel impregnation and carbothermal reduction [31]. This study investigated the feasibility of using bamboo to prepare biomorphic porous silicon carbide (bio-SiC) ceramics through a combination of sol-gel impregnation and carbothermal reduction. The effects of sintering temperature, sintering duration, and sol–gel impregnation cycles on the crystalline phases and microstructure of bio-SiC were investigated. X-ray diffraction patterns revealed that when bamboo charcoal-SiO_2_ composites (BcSiCs) were sintered at 1700 °C for more than 2 h, the resulting bio-SiC ceramics exhibited significant SiC diffraction peaks. In addition, when the composites were sintered at 1700 °C for 2 h, scanning electron microscopy micrographs of the resulting bio-SiC ceramic prepared using a single impregnation cycle showed the presence of SiC crystalline particles and nanowires in the cell wall and cell lumen of the carbon template, respectively. However, bio-SiC prepared using three and five repeated cycles of sol-gel impregnation exhibited a foam-like microstructure compared with that prepared using a single impregnation cycle. Similar results were observed for the bio-SiC ceramics prepared from bamboo-SiO_2_ composites (BSiCs). Accordingly, bio-SiC ceramics can be directly and successfully prepared from BSiCs, simplifying the manufacturing process of SiC ceramics.

The fast growing wood polymer composites (WPC) sector, and the closely related natural fiber composites (NFC) sector, presents many new opportunities for utilizing wood as well as natural fibers or agricultural residues, as filler or reinforcement in polymer profiles and moldings. The field combines wood processing techniques for fiber or filler preparation with polymer science and engineering, and a range of polymer processing techniques including extrusion, injection molding, compression molding, and pultrusion. Wood plastic composites (WPCs) incorporating graphene nano-platelets (GNPs) were fabricated using hot-pressed technology to enhance thermal and mechanical behavior [32]. The influences of thermal filler content and temperature on the thermal performance of the modified WPCs were investigated. The results showed that the thermal conductivity of the composites increased significantly with the increase of GNPs fillers, but decreased with the increase of temperature. Moreover, thermogravimetric analysis demonstrated that coupling GNPs resulted in better thermal stability of the WPCs. The limiting oxygen index test also showed that addition of GNPs caused good fire retardancy in WPCs. Incorporation of GNPs also led to an improvement in mechanical properties as compared to neat WPCs. Through a series of mechanical performance tests, it could be concluded that the flexural and tensile moduli of WPCs were improved with the increase of the content of fillers.

A novel wood-plastic composite (WPC) lumber has shown potential to replace high-density polyethylene (HDPE) lumber in the construction of aquacultural geodesic spherical cage structures [33]. Six HDPE and six WPC assemblies, which are representative of typical full-size cage dimensions, were fabricated by bolting pairs of triangular panel components made with connected struts. Half of the panel assemblies had a plastic-coated steel wire mesh to simulate the actual restraint in field applications of the cages. The objective of the research was to characterize the structural performance of the panel assemblies under compressive loading. To determine the critical buckling load for the panel assemblies made from WPC and HDPE struts with and without wire mesh, Southwell’s method was implemented. A two-dimensional (2D) linear finite element analysis model was developed to determine axial forces in the struts of the panel assembly for the applied load and boundary conditions. This model was used to determine strut compressive forces corresponding to the Southwell’s method buckling load and the experimental failure load. It was found that the wire mesh increased the load capacity of both HDPE and WPC panel assemblies by a factor of two. The typical failure mode of the panels made from HDPE lumber struts, with and without wire mesh, was buckling of the struts, whereas the failure mode of the WPC panels, with and without wire mesh, was fracture at the notched section corresponding to the location of the bolts. The load capacity of the panel assemblies made from WPC lumber struts was three times and 2.5 times higher than the load capacity of the panel assemblies made from HDPE lumber struts with and without wire mesh, respectively.

Virgin thermoplastics, such as polypropylene (PP), polyethylene (PE), polystyrene (PS), and polyvinyl chloride (PVC), are commonly used as matrices for manufacturing WPCs [with wood flour, but using recycled plastic as a matrix was only reported in a few studies [34]. A paper in this Special Issue examined the effects of selected types of thermoplastics on the physical and mechanical properties of polymer-triticale boards [35]. The investigated thermoplastics differed in their type (polypropylene (PP), polyethylene (PE), polystyrene (PS)), form (granulate, agglomerate) and origin (native, recycled). The resulting five-ply boards contained layers made from different materials (straw or pine wood) and featured different moisture contents (2%, 25%, and 7% for the face, middle, and core layers, respectively). Thermoplastics were added only to two external layers, where they substituted 30% of straw particles. This study demonstrated that, irrespective of their type, thermoplastics added to the face layers most favorably reduced the hydrophobic properties of the boards, i.e., thickness, swelling, and V100, by nearly 20%. The bending strength and modulus of elasticity were about 10% lower in the experimental boards than in the reference ones, but still within the limits set out in standard for P7 boards according to EN 312.

As a conclusion or a general remark regarding the future of wood composites, it can be said that the ability of wood composites to be tailored to specific uses, together with their strength properties and affordability, makes them a viable solution to reducing the need for solid wood. They have been successfully applied in all forms of building, from small home projects to industrial construction work, and as technology surrounding their manufacture only advances, the future looks bright.
